# Canine leishmaniasis in Southern Italy: a role for nitric oxide released from activated macrophages in asymptomatic infection?

**DOI:** 10.1186/1756-3305-1-10

**Published:** 2008-05-09

**Authors:** Maria A Panaro, Olga Brandonisio, Donato de Caprariis, Pasqua Cavallo, Antonia Cianciulli, Vincenzo Mitolo, Domenico Otranto

**Affiliations:** 1 Department of Human Anatomy and Histology, Medical School, University of Bari, Policlinico, Italy; 2 Department of Internal Medicine, Immunology and Infectious Diseases, Medical School, University of Bari, Policlinico, Italy; 3 Department of Animal Health and Welfare, Faculty of Veterinary Medicine, University of Bari, Valenzano (BA), Italy; 4 Department of Veterinary Public Health, Faculty of Veterinary Medicine, University of Bari, Valenzano (BA), Italy

## Abstract

**Background:**

Human and canine leishmaniasis (CanL) by *Leishmania infantum *is endemic in Italy, with a high percentage of infected asymptomatic animals. However, the immune response mechanisms underlying the clinical presentation of CanL have not been fully investigated. Among leishmanicidal molecules produced by activated macrophages, nitric oxide (NO) produced by an inducible NO synthase seems to play an important protective role, but no conclusive data are available. Therefore, NO released by cultured macrophages from dogs with natural *Leishmania *infection living in an endemic area for CanL was evaluated.

**Methods:**

On the basis of one year's clinical and laboratory follow-up, 22 dogs infected by *Leishmania infantum *were identified and grouped as: asymptomatic dogs (n = 13) and dogs with symptoms of leishmaniasis (n = 9). Each animal was bled twice at 4-month intervals and macrophage and lymphocyte cultures were obtained from peripheral blood mononuclear cells. Supernatants of *L. infantum*-infected macrophage cultures, with or without addition of autologous lymphocytes, were assayed for NO production by Griess reaction for nitrites.

**Results:**

In the first months of the infection the levels of NO in supernatants of *Leishmania*-infected macrophages were higher in symptomatic than in asymptomatic dogs, but they were significantly increased in the latter group eight months after the diagnosis of infection. Furthermore, NO release significantly decreased in the presence of autologous lymphocytes in both groups of animals.

**Conclusion:**

These results suggest that NO may be involved in the long-term protection of dogs against natural *Leishmania *infection and in the clinical presentation of canine leishmaniasis in the Mediterranean area.

## Background

Protozoa of the *Leishmania *genus infect mononuclear phagocytes of several mammalian species, including dogs, in which they often give rise to a chronic, non self-healing visceral disease [1]. The dog is the main competent and reservoir host for *Leishmania infantum *in the Mediterranean Basin, Middle East and Central Asia up to Pakistan and China, of *L. chagasi *in Central and South America and of *L. peruviana *in the Peruvian Andes [2,3]. In addition, its role as a reservoir was indicated for *L. donovani *in Eastern Sudan and Morocco, and for *L. tropica *in Iran and probably in Northern Africa [4]. Human leishmaniasis has recently been categorised as an emerging and uncontrolled disease (i.e. category 1), with a total disease burden of 2,090,000 Disability-Adjusted Life-Years (DALYs) and 51,000 deaths per year [5].

Human and canine leishmaniasis (CanL) from *Leishmania infantum *zymodeme MON-1 is endemic in Central and Southern regions of Italy as well as the islands [6]. In recent years, a rapid increase of autochthonous cases of CanL has been recorded also in Northern Italian regions as a consequence of the spread of suitable habitats for the sandfly vector [7]. In the Apulia region (Southern Italy) a yearly incidence rate of CanL of about 9.52% has been estimated in both farm and kennel dogs [8], with a high percentage (53.1%) of serologically positive asymptomatic animals [9]. In fact, these dogs display either asymptomatic or symptomatic parasitism, the latter ranging from limited to multifocal tissue damage with or without hypergammaglobulinaemia. Transmission of *Leishmania *spp. occurs when phlebotomine sandfly vectors feed on infected dogs, either symptomatic or asymptomatic [10], so both groups of animals are significant for the transmission of human and CanL. This highlights the importance of dogs in spreading human disease, also in the light of a possibly prolonged subpatent period [11].

Control measures for CanL include evaluation of new diagnostic tests [12], reliable screening of new compounds for chemotherapy [13], zoonotic reservoir control, including insecticide-based preparations for dog protection [14,15], and development of an affordable and effective vaccine for dogs [reviewed by [1]]. Under these circumstances, a knowledge of the immune mechanisms involved in animal protection plays a pivotal role in understanding the pathogenesis and clinical progression of the disease, as well as in the development of vaccines. Among leishmanicidal molecules produced by interferon (IFN)-γ-activated macrophages, nitric oxide (NO) produced by an inducible NO synthase (i.e. iNOS or NOS2) seems to play an important protective role [16], which is of interest also for CanL. Indeed, dog macrophages infected *in vitro *by *L. infantum *promastigotes produced NO after stimulation with cytokine-enriched peripheral blood mononuclear cell (PBMC) supernatants [17] and expressed NOS2, after stimulation with IFN-γ and bacterial lipopolysaccharide (LPS) [18]. However, the immune response mechanisms underlying the clinical presentation of CanL (i.e. in asymptomatic and symptomatic animals) have not been fully investigated and no conclusive data are available concerning the role played by NO in this context. Moreover, data on NO release by macrophages available from laboratory investigations on a murine model [19] may be quite different from what happens under field conditions in naturally infected dogs.

This study has investigated for the first time if NO released by activated macrophages from naturally infected dogs, estimated by the nitrite content in the culture supernatant, may play a role in the asymptomatic parasitism displayed by dogs in Southern Italy.

## Methods

### Animal sampling and clinical examination

The animals came from two kennels in the Apulian region, southern Italy (latitude 42° and 39° North, longitude 15° and 18° East), where endemic CanL had been reported over the previous years [8]. A total of 22 dogs that were negative for *L. infantum *(at parasitological and serological tests, see below) in March 2005 but resulted newly infected by *L. infantum *in March 2006 (after one sandfly season) were enrolled in the study. All selected animals were positive at parasitological (i.e. PCR on skin biopsy and/or microscopic examination of lymph node smears) and/or serological tests for *Leishmania *[15] but did not exhibit clinical symptoms or laboratory abnormalities of leishmaniasis.

During follow-up, clinical examination of dogs was performed monthly, in order to evaluate a possible disease progression. At 4 and 8 months follow-up visits from the first diagnosis of *Leishmania *infection (i.e. July and November 2006), 20 mL heparinized peripheral blood samples were obtained from each dog. Animals were handled and sampled with the owners' consent and approval by the Ethics Committee of the University of Bari.

### Parasites

Parasites were isolated from the bone marrow of a *Leishmania*-infected dog and cultured on Tobie-Evans medium at 24°C. The isolated strain was typed by the Istituto Superiore di Sanità (Rome, Italy) and belonged to the *L. infantum *species, zymodeme MON1. Promastigotes at day 4 of culture were used, since the maximum percentage of metacyclic (virulent) promastigotes is detected on the 4^th ^day of growth for *Leishmania infantum *[20]. The liquid phase of the tubes was collected and centrifuged at 350 g for 10 min. Supernatants were then discarded and the pellets were suspended in phosphate buffered saline (PBS), pH 7.2 and washed 3 times by centrifugation at 350 g for 10 min. Finally, promastigotes were counted after immobilization by 2–3 drops of 70% ethanol.

### Isolation of peripheral blood mononuclear cells (PBMC)

PBMC were isolated from 20 mL of heparinized peripheral blood, diluted with sterile Hanks' balanced salt solution (HBSS; Gibco-Invitrogen, Carlsbad, CA, USA) at a 1:1 (v/v) ratio and centrifuged on the cell separation medium Lympholyte-H (CEDARLANE, Burlington, Ontario, Canada) at 700 g for 15 min at room temperature. Then, isolated PBMC were extensively washed and suspended in RPMI 1640 complete medium, supplemented with 2 mM glutamine, 10% heat-inactivated (56°C for 30 min) foetal calf serum (FCS), 100 μg/mL streptomycin and 100 IU/mL penicillin. Cell viability was evaluated by trypan blue dye exclusion and resulted greater than 98%. Isolated cells were cultured at a density of 1 × 10^6^/mL in 4-well microculture plates (Nunc, Roskilde, Denmark), at 37°C, 5% CO_2 _for 24 h to permit monocyte adherence. Lymphocytes-enriched supernatants were then collected and cultured in complete RPMI.

### Macrophage culture, infection and treatment procedures

After 24 h of incubation, adherent mononuclear cells (1 × 10^6^/mL for each well) were washed-out three times with HBSS and then incubated in fresh complete medium for 8 days to permit the differentiation of monocytes into macrophages. During this period the medium was replaced every 48–72 h. Cell viability determined by trypan blue exclusion was greater than 98%. Morphological features such as increased size, the presence of short, blunt pseudopodia and non specific esterase staining showed that more than 96% of the cells were macrophages.

After 8 days of adherence, macrophages were counted by direct immunofluorescence for the CD16 macrophage marker, using a FITC-labelled anti-CD16 monoclonal antibody (Becton-Dickinson, USA), and 2,5 × 10^5^CD16+ cells were infected with living *L. infantum *promastigotes (1:3 cell/parasite ratio). After 24 h, infected macrophages were gently washed to remove non-ingested parasites, monitored by FITC-labelled anti-CD16 monoclonal antibody to ensure that no macrophages were lost when washing the culture and then cultured for 48 h, in the presence or absence of autologous lymphocytes (5 × 10^5^/mL for each well), which were separately cultured – once depleted of adherent cells. Untreated uninfected macrophages were used as controls. In preliminary experiments the number of macrophages engulfing parasites was evaluated. For this assay, macrophages (1 × 10^6^/mL) were incubated at 37°C in a 5% CO_2 _humidified atmosphere with metacyclic *Leishmania *promastigotes at a 1:3 cell/parasite ratio for 48 h. At the end of the incubation periods, cells were collected from the plates by detachment after the addition of cold (+4°C) medium and vigorous aspiration. Cells were then washed with PBS pH 7.2, cytocentrifuged onto glass slides and stained with methanol-Giemsa stain. The phagocytosis percentage of macrophages was microscopically calculated by counting the percentage of cells containing at least one amastigote/300 cells and resulted 84.6 ± 0.9, being the mean ± SD of three separate experiments.

### NO production evaluation

The concentration of nitrite (NO2-) released by macrophages determined by the Griess reaction was used as an indicator of NO production. Briefly, after 72 h of infection, cell culture supernatants from macrophages were mixed with an equal volume of Griess reagent, as described by Ding and colleagues [21]. Experiments were also performed in the presence of 200 μM L-NG-monomethylarginine (L-NGMMA, Sigma-Aldrich), a competitive inhibitor of the iNOS, in order to assess whether NO production is dependent upon iNOS activation. The absorbance was spectrophotometrically measured at 540 nm and the NO2- concentration was determined by a standard curve of NaNO2 and expressed as nmol/mL. To avoid interference by any nitrites present in the medium, the medium employed for the macrophage cultures was used as blank.

### Statistical analysis

Results were statistically examined by analysis of variance (one-way ANOVA) and a *p*-value < 0.05 was considered statistically significant.

## Results

**Figure 1:**
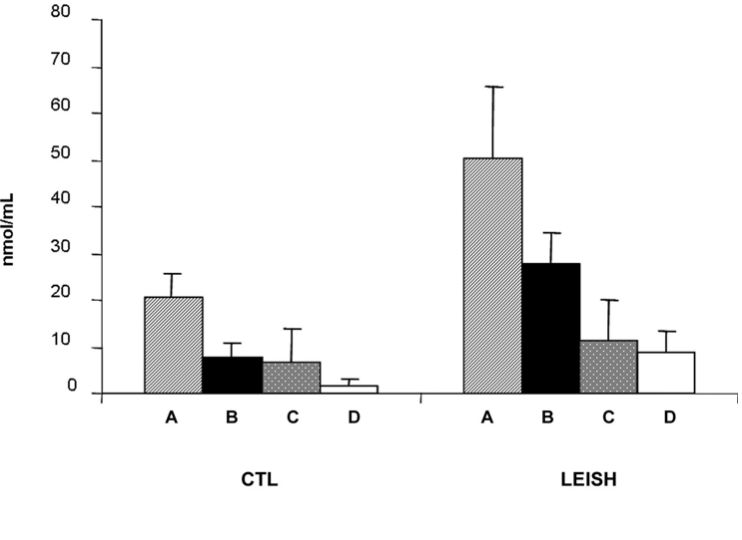
**NO release by monocyte-derived macrophages four months after the diagnosis of *Leishmania *infection**. **CTL**: uninfected macrophages (controls). **LEISH**: *L. infantum*-infected macrophages. **A**, macrophages from symptomatic dogs incubated without lymphocytes; **B**, macrophages from symptomatic dogs incubated in presence of autologous lymphocytes; **C**, macrophages from asymptomatic dogs incubated without autologous lymphocytes; **D**, macrophages from asymptomatic dogs incubated with autologous lymphocytes. Results are expressed as means ± SE.

Of the 22 infected dogs initially tested, 13 remained asymptomatic at four and eight months of follow-up, while 9 showed one or more symptoms of leishmaniasis (i.e. dermatitis, lymphadenopathy, conjunctivitis, skin ulcers) and/or hypergammaglobulinemia. At the first follow up (i.e. after four months from the first diagnosis of *Leishmania *infection), PBMC-derived macrophages infected *in vitro *by *L. infantum *produced a significantly higher amount of NO (p < 0.001) than macrophages not infected *in vitro*, used as controls. In addition, the levels of NO in supernatants of *Leishmania*-infected macrophages were significantly higher (p < 0.001) in symptomatic *vs *asymptomatic dogs (Fig. 1). Moreover, the addition of autologous lymphocytes significantly (p < 0.001) decreased NO production only in symptomatic dogs.

**Figure 2:**
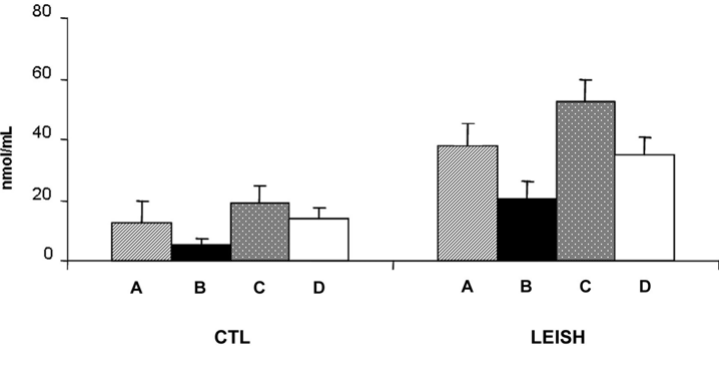
**NO release by monocyte-derived macrophages eight months after diagnosis of *Leishmania *infection**. **CTL**: uninfected macrophages (controls). **LEISH**: *L. infantum*-infected macrophages. **A**, macrophages from symptomatic dogs incubated without lymphocytes; **B**, macrophages from symptomatic dogs incubated with autologous lymphocytes; **C**, macrophages from asymptomatic dogs without lymphocytes; **D**, macrophages from asymptomatic dogs with autologous lymphocytes. Results are expressed as means ± SE.

On the contrary, at the second follow-up (i.e. eight months from the diagnosis of *Leishmania *infection), NO released from *Leishmania*-infected macrophages, was significantly higher (p < 0.001) in asymptomatic *vs *symptomatic dogs (Fig. 2). As in the samples taken after 4 months, this production was significantly (p < 0.001) lower in supernatants from uninfected macrophages used as control. Results from macrophages co-cultured with autologous lymphocytes showed that NO production was significantly reduced in *Leishmania-*infected macrophages both in asymptomatic and symptomatic dogs (*p *< 0.001 and *p *< 0.05, respectively) (Fig. 2).

**Figure 3:**
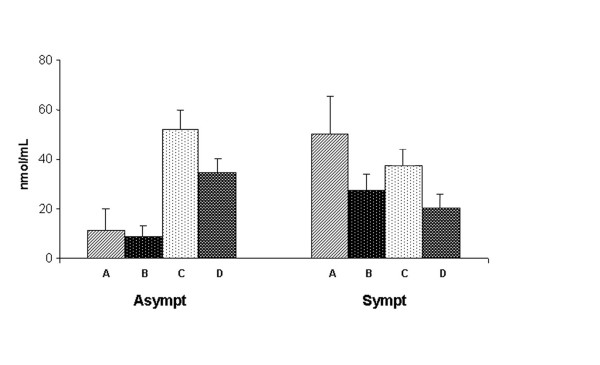
**NO release by *L. infantum*-infected macrophages from asymptomatic (left) and symptomatic (right) dogs at four and eight months after diagnosis of *Leishmania *infection**. **A**, macrophages without lymphocytes (4 months); **B**, macrophages incubated with autologous lymphocytes (4 months); **C**, macrophages without lymphocytes (8 months); **D**, macrophages with autologous lymphocytes (8 months). Results are expressed as means ± SE.

In order to investigate a possible variation in NO production during the course of the infection, results obtained after four and eight months were compared. Figure 3 shows that in infected macrophage supernatants from asymptomatic dogs, NO production was significantly increased (*p *< 0.001) after eight months, in comparison with samples taken after four months. Also in these experiments, co-incubation of infected macrophages with autologous lymphocytes significantly decreased NO production. Interestingly, in dogs that developed symptoms, no increase in NO production by infected macrophages was observed in samples obtained after eight months in comparison with those taken after four months from diagnosis of the infection.

Finally, macrophage stimulation in the presence of the L-arginine analogue NO synthase inhibitor (L-N^G^MMA) caused a reduction of NO production at basal levels, thus confirming that NO production in infected cells was induced by iNOS activation (data not shown).

## Discussion

The NO production by cultured PBMC-derived macrophages has been investigated herein for the first time in both symptomatic and asymptomatic dogs with natural *Leishmania *infection. Results of this study demonstrated that: i) NO release from macrophages was enhanced by *in vitro *infection; ii) in the first months of the infection the levels of NO in supernatants of *Leishmania*-infected macrophages were higher in symptomatic than in asymptomatic dogs, but significantly increased in the latter group after eight months from the diagnosis of *Leishmania *infection; iii) co-culture of macrophages with autologous lymphocytes reduced NO production especially in symptomatic dogs.

In the light of these results, it is possible that cells obtained from symptomatic animals are pre-activated *in vivo*, and hence that infection of these cells *in vitro *provides a secondary stimulus that results in NO release. According to this reasoning, NO production at the beginning of infection might be an indicator of immune activity in infected animals but not an indicator of resistance. However, the higher levels of NO observed in follow-up in symptom-free animals may also suggest a protective role for this molecule in the long term asymptomatic parasitism displayed by dogs in Southern Italy. The augmented NO production in these animals may be due to cytokines such as IFN-γ and tumor necrosis factor (TNF)-α, which exert an activating effect on iNOS and regulate the enzyme at both the transcriptional and post-transcriptional levels [22].

The involvement of NO in protecting against CanL has already been demonstrated by inducing antileishmanial macrophage activity via the L-arginine NO pathway [23]. In addition, an increased NO production and anti-leishmanial activity of infected cells was demonstrated using canine macrophages activated by a supernatant derived from a *Leishmania*-specific T cell line which contained IFN-γ, TNF-α and IL-2 [24]. The role played by NO in parasitocidal activity has also been investigated in the context of vaccination studies. It was found that the amount of IFN-γ in dog PBMC supernatants and NO production by macrophages increased after administration of a killed *L. infantum *promastigote vaccine [25]. In another study, vaccine-induced protection correlated with a Th1-type cellular immune response specifically directed against purified excreted-secreted antigens from *L. infantum *promastigotes (LiESAp), including: i) an enhanced NO-mediated anti-leishmanial activity of dog macrophages co-cultured with IFN-γ producing autologous lymphocytes [26] and ii) NO-mediated apoptosis of intracellular amastigotes in canine macrophages [27]. The above results have also been confirmed under field conditions, with a significantly enhanced NO-mediated anti-leishmanial macrophage activity almost exclusively revealed in vaccinated animals [28]. In a separate study carried out in Brazil, PBMC supernatants from dogs immunized with promastigote lysates and infected with *L. chagasi *were also able to stimulate the parasitocidal activity of macrophages in healthy dogs, through NO production [29]. Several cellular targets may be subject to NO toxicity in *Leishmania *parasites, including enzymes of glycolysis and respiratory metabolism, as well as trans-membrane transport systems [30].

Moreover, exposure of amastigotes to moderate concentrations of donor NO or to endogenous NO produced by macrophages resulted in cell death, with extensive fragmentation of nuclear DNA, in both axenic and intracellular amastigotes of *L. amazonensis *[31]. NO is also involved in the parasitocidal effect of anti-*Leishmania *drugs. In fact, sodium antimony gluconate treatment induces murine macrophages activation of the p38 MAPK signalling pathway, with subsequent TNF-α release, NO production and NO-dependent parasite killing [32]. Moreover, SbIII-resistant amastigotes were also resistant to NO toxicity when delivered extracellularly via NO donors or intracellularly via macrophage activation [33].

In the present study addition of autologous lymphocytes reduced NO production in co-cultured macrophages. Which cell fraction of PBMCs was responsible for this suppression is an area requiring further investigation, also using commercially available dog CD markers. Moreover, the decreased NO release in the presence of autologous lymphocytes suggests a likely role for anti-inflammatory cytokines (e.g. IL-4, IL-10, IL-13) and transforming growth factor (TGF)-β, released by lymphocytes of *Leishmania *infected animals, whose inhibitory effect on signal transduction for iNOS and NO production was previously described in macrophages and other cells in different animal models [22,34]. Although the relationship between anti-inflammatory cytokines and NO production in dogs deserves further investigation, the production of these cytokines in canine *Leishmania *infection has already been demonstrated, like TGF-β1 and IL-10 in the spleen and liver of both symptomatic and asymptomatic dogs naturally infected by *L. chagasi *[35], and IL-4 expression in skin biopsies [36] and in spleen [37] of *L. infantum*-infected dogs.

## Conclusion

Overall, the results of this paper suggest that: i) NO could be strongly involved in the long-term protection of dogs against natural *L. infantum *infection and in the clinical presentation of leishmaniasis in the Mediterranean area, and ii) autologous lymphocytes may exert an inhibitory effect on NO production by macrophages from naturally-infected animals. These results may contribute to a better understanding of the pathogenesis and clinical course of CanL in endemic areas, helping to develop control strategies both for human and canine leishmaniasis.

## Competing interests

The authors declare that they have no competing interests.

## Authors' contributions

PMA conceived of the study, participated in its design and coordination and helped to draft the manuscript. BO participated in the study design and drafted the manuscript. dCD participated in animal sampling and clinical examination. CP carried out cell culture studies. CA participated in cell culture experiments and performed the statistical analysis. MV participated in the study design and coordination. OD participated in the study design and coordination and helped to draft the manuscript. All authors read and approved the final manuscript.
